# Evaluation of high-fidelity simulation training in radiation oncology using an outcomes logic model

**DOI:** 10.1186/1748-717X-9-189

**Published:** 2014-08-28

**Authors:** Meredith Giuliani, Caitlin Gillan, Olive Wong, Nicole Harnett, Emily Milne, Doug Moseley, Robert Thompson, Pamela Catton, Jean-Pierre Bissonnette

**Affiliations:** Radiation Medicine Program, Princess Margaret Cancer Centre, Toronto, Canada; Department of Radiation Oncology, University of Toronto, 610 University Avenue, Toronto, ON M5G 2 M9 Canada

**Keywords:** Simulation, Radiation oncology, Residency, Postgraduate, High-fidelity, Interprofessional practice, Teamwork, Radiation therapy

## Abstract

**Purpose:**

To evaluate the feasibility and educational value of high-fidelity, interprofessional team-based simulation in radiation oncology.

**Methods:**

The simulation event was conducted in a radiation oncology department during a non-clinical day. It involved 5 simulation scenarios that were run over three 105 minute timeslots in a single day. High-acuity, low-frequency clinical situations were selected and included HDR brachytherapy emergency, 4D CT artifact management, pediatric emergency clinical mark-up, electron scalp trial set-up and a cone beam CT misregistration incident. A purposive sample of a minimum of 20 trainees was required to assess recruitment feasibility. A faculty radiation oncologist (RO), medical physicist (MP) or radiation therapist (RTT), facilitated each case. Participants completed a pre event survey of demographic data and motivation for participation. A post event survey collected perceptions of familiarity with the clinical content, comfort with interprofessional practice, and event satisfaction, scored on a 1–10 scale in terms of clinical knowledge, clinical decision making, clinical skills, exposure to other trainees and interprofessional communication. Means and standard deviations were calculated.

**Results:**

Twenty-one trainees participated including 6 ROs (29%), 6 MPs (29%), and 9 RTTs (43%). All 12 cases (100%) were completed within the allocated 105 minutes. Nine faculty facilitators, (3MP, 2 RO, 4 RTTs) were required for 405 minutes each. Additional costs associated with this event were 154 hours to build the high fidelity scenarios, 2 standardized patients (SPs) for a total of 15.5 hours, and consumables.The mean (±SD) educational value score reported by participants with respect to clinical knowledge was 8.9 (1.1), clinical decision making 8.9 (1.3), clinical skills 8.9 (1.1), exposure to other trainees 9.1 (2.3) and interprofessional communication 9.1 (1.0). Fifteen (71%) participants reported the cases were of an appropriate complexity. The importance of further simulation events was rated highly at 9.1/10.

**Conclusions:**

High-fidelity simulation training is feasible and effective in a radiation oncology context. However, such educational activities require significant resources, including personnel and equipment.

## Introduction

The rapid evolution in technology and clinical practice in radiation oncology has created a significant challenge for postgraduate training programs [1]. Traditional educational and assessment techniques such as lectures, clinical observerships, written examinations and objective structured clinical exams (OSCEs) are not independently adequate to address modern competencies with respect to interprofessional education and practice (IPE, IPP) [2], team-based competencies [3], and imaging literacy [1].

Simulation-based training is widely used in surgical specialties and emergency medicine training programs to master techniques requiring motor skills [4, 5] and more recently to address team-based competencies [6]. Medical professionals traditionally receive ‘siloed’ training, which reward individual accomplishments, [7] and simulation is an effective method to changing both behaviors and attitudes towards improving team-based competencies [6]. To date, high-fidelity simulation has not been described in radiation oncology training programs.

The purpose of this study was to evaluate the feasibility and educational outcomes of high-fidelity, interprofessional team-based simulation training in radiation oncology using an outcomes-logic-model.

## Methods

### Simulation event

For the purposes of this work ‘simulation’ refers to ‘simulation-based training’ for educational purposes. An interprofessional research team, consisting of radiation oncologist (RO), medical physicist (MP) and radiation therapist (RTT) educators developed 5 simulation scenarios that reflected high-acuity, low-frequency clinical situations that the study investigators thought might be best learned in a high-fidelity simulation environment. High-acuity refers to clinical situations requiring significant and often rapid medical decision. High-fidelity refers to scenarios which most closely re-create actual clinical encounters. A simulation event was conducted in a radiation oncology department during a non-clinical day. Facilitators, either radiation oncologists or radiation therapists, conducted each case with an interprofessional trainee team.

### Participants

The study was designed to suit a convenience sample of trainees from RO, MP and RTT training programs from one academic institution involving 10 affiliated clinical sites. Radiation oncology is a 5 year training program [8] and trainees from any year were eligible to participate. MP is a two year training program [9] and trainees from any year were eligible to participate. The RTT program is a 3 year program [10]; however, due to its structure, only students in their third year of training were eligible to participate.

All eligible trainees (n = 67; 42 RTT, 10 MP, 15 RO), were approached via an email sent by a Research Assistant (RA), two weeks prior to the event. Written consent for participation was obtained. This study was conducted with research ethics board approval.

### Outcomes logic model

An outcomes-logic-model was used to document the extent to which the simulation event attained its objectives. The outcomes-logic-model was selected as it allows a comparison among the resources required for a program, the activities of the program and the results or outcomes [11]. The objectives, based on various program components, were selected by the research team as those that were predictive of desired feasibility and educational outcomes. Each outcome was paired with a measurement indicator to determine the degree to which the objective has been achieved. All measurement indicators were designed to be measurable characteristics or specific statistics (such as numbers or percentages) with baseline targets. Immediate outcomes are those that occur during or immediately after completion of the event [11].

## Results

### Program inputs

Faculty RO, MP and RTTs were required to conduct this event and serve as case and group facilitators. Nine faculty members, excluding the study investigators, filled these roles and each committed 45 minutes to orientation training, 315 minutes to conducting the cases and 45 minutes to debriefing. A minimum of 20 trainees were required to conduct the event; 5 RO residents, 5 MP residents and 10 RT students to create 5 teams consisting of 1 RO, 1MP and 2 RTTs. Each student required 8 hours to attend the event. In addition, three authorized users were needed for 16 hours to operate the clinical equipment, as per regulations of the Canadian Nuclear Safety Commission (8 hours preparation time before the event and 8 hours of the event).

This simulation event required access to a clinical radiation oncology department with an available linear accelerator, Elekta Inc. XVI © cone beam computed tomography (CBCT) image registration software, brachytherapy after-loaders and a CT simulator for 8 hours. The linear accelerator circuit breaker was switched off to ensure participant safety by preventing radiation generation while allowing all motorized motions. The event required access to 5GB of data storage.

The main costs associated with this event were human and physical resources (both enduring and consumable). An education assistant was needed to create the high fidelity scenarios and two standardized patients (SPs) who were actors were required on the event day to participate in two cases (brachytherapy and electron cases). The educational assistant committed 154 hours to the event in advance to prepare the scenarios. A detailed description of these activities is available in Table 1. The total time for SP training and performance in the simulations was 15.5 hours. Printed materials were required for the candidates in each case and to perform assessments. Make-up and fake skin were required to simulate the scalp tumor on the standardized patient. For the brachytherapy case, a decommissioned remote after-loader with cables and dummy radioactive source, catheter channels, tandem & ring were used. Unfortunately, one of the catheter channels was damaged during the event. Prior to the event it was no longer in clinical use but required replacement for future simulation events. A full list of required equipment can be found in Table 2.

**Table 1:** **Preparation activities for individual high-fidelity cases**

Case	Hours	Case building requirements
Pediatric	32	- Retrieval of case information (CT, MRI, EPIs taken on treatment,)
		- Anonymization of case information
		- Creation of patient in simulation radiation record (diagnosis, prescriptions, notes, images, consent)
		- Collecting materials (calipers, simulation doll, nasal prongs, calculator, pens, acetate, bolus, calculation data book, blank manual calculation sheets)
		- Set up of simulation treatment room, including loading of anonymized CT/MRI
Electrons	32	- Retrieval of case information (clinical notes, pathology report)
		- Anonymization of case information, mosaiQ^TM^ notes, EPR notes
		- Creation of patient into simulation radiation record (diagnosis, prescriptions, notes, images, consent)
		- Creation of standardized patient script
		- Hiring of standardized patient
		- Collecting materials (neck rests, prone medullaboard, prone pillow, electron applicators and inserts, rulers, bolus, tape, Saran wrap^TM^, multiple neck rests, acetate, markers, pens, calculators, data-books, PDD tables)
		- Set up of simulation treatment room
CBCT	15	-Retrieval of case information (Diagnostic CT, clinical photo from nasopharnygoscopy)
		-Retrieval of planning CT with artifact, GTV, CTV, PTV contours in Pinnacle
		-Retrieval of CBCT with day 1 match XVI and day 13 match XVI
		-Anonymization of all patient information collected
		-Creation of patient into simulation radiation record (diagnosis, prescriptions, notes, images, consent)
		-Loading of XVI images into multiple computer stations
Lung SBRT	15	-Retrieval of case information (diagnostic PET, diagnostic CT, EMR report, Simulation 4D CT images from maximum inhale, maximum exhale, average, and MIP phases)
		- Anonymization of all patient information collected
		-Creation of patient into simulation radiation record (diagnosis, prescriptions, notes, images, consent)
		-Loading of treatment planning information into simulation system
Brachytherapy	60	- Recording treatment during clinical hours for audio input
		- Creation of Powerpoint slides to replicate clinical interface of treatment unit
		- Collecting materials to transport (pig, stirrups, transfer tubes, dose rate meters, decommissioned remote after loader with cables and dummy source, zoe phantom, catheter channels, tongs, forceps, timer)
		- Transferring of decommissioned remote after loader
		- Creation of replica control console
		- Set up of simulation treatment room

**Legend:** CT – computed tomography; MRI – magnetic resonance imaging; EPI – electronic portal image; GTV- gross tumor volume; CTV – clinical target volume; PTV – planning target volume; PDD – percent depth dose; EPR – electronic patient record; CBCT – cone beam CT; PET – positron emission tomography. SBRT - stereotactic Boday Radiotherapy. MIP - maximum intensity projection.

**Table 2:** **Program components and descriptions**

Component	Description
**Inputs:** What resources were dedicated to or consumed by the program?	● Faculty time
	● Trainee time
	● Facility space
	● Data storage
	● **Non-consumed equipment**
	○ Linear Accelerator
	○ XVI software
	○ Pinnacle Treatment planning system
	○ Data book for different energies
	○ Calculators
	○ Simulation infant-doll
	○ Caliper
	○ Camera (for treatment setup photo)
	○ Nasal prongs
	○ Multiple neck rests
	○ Prone pillow
	○ Electron applicators and inserts
	○ Tantalum
	○ Decommissioned remote afterloader with cables and dummy source, catheter channels, tandem, & ring
	○ Pig
	○ Tongs
	○ Radiation survey meter
	○ Radiation check source
	○ Console mock up
	○ Laptop for “treatment”
	○ Timer
	○ Female pelvis phantom
	○ Radiation safety officer contact
	**● Consumed equipment**
	○ Manual calculation sheets
	○ Pencils
	○ Acetate
	○ Tape
	○ Bolus 0.5 cm
	○ Bolus 1.0 cm
	○ Markers
	○ Packing
	○ Suture removal kit
	○ Incident report forms
**Activities:** What does the program do with the inputs to fulfill its mission?	● Create high fidelity case scenarios
	● Train standardized patients for cases
	● Create assessment tools for medical expertise, team function and interprofessional knowledge/perceptions
**Outputs:** What are the direct outputs of the program activities?	● Completion of the simulation program with pre and post event assessments
	● Number of participants (by discipline)
	● Number of facilitators (by discipline)
	● Perception of clinical fidelity
	● Participant satisfaction
**Outcomes:** What are the immediate and intermediate benefits for participants during and after the program?	**Immediate:**
	● Increase medical knowledge
	● Improved decision making skills
	● Change in perception of interprofessional roles
	● Participant exposure to a unique educational opportunity

### Program activities

Five high fidelity simulation scenarios were developed and implemented with a group of interprofessional trainees from RO, MP and RTT including high dose rate (HDR) brachytherapy ‘stuck source’ emergency, 4D-CT lung stereotactic body radiotherapy (SBRT) simulation artifact, pediatric emergency clinical mark-up, electron scalp irradiation trial set-up, and a larynx cone beam CT (CBCT) image mis-registration incident. Assessment tools were created to determine the event’s impact on participants’ familiarity with the clinical content, perceptions of interprofessional practice, and perceptions of the event. To assess participant satisfaction, participants reported on a 1–10 Likert scale willingness to participate in a subsequent high-fidelity simulation event. To determine participants’ perception of the value of the event to enhance 1) their exposure to other trainees, 2) interprofessional communication and 3) their clinical knowledge, decisions making and clinical skills they reported on a 1–10 Likert scale (10 = extremely valuable). Participants reported how prepared they were to manage each case based on their current training on a 1 to 10 scale (10 = fully equipped). Participants then reported how important they believe exposure to each case in training was on a 1 to 10 scale (10 = very important). Finally, participants reported the value of the team interaction to delivering patient care on scale of 1 to 10 (10 = very important), as an element of the Trainee Test of Team Dynamics (TTTD) [12]. Participants also completed the validated Readiness for Interprofessional Learning Scale (RIPLS) which has 19 5-point Likert scale items and higher scores indicate positive attitudes with respect relating to teamwork and professional identity/ responsibilities [13], the UWE Entry Level Interprofessional Questionnaire (UWEIQ) which is a 27-item, 4- or 5-point Likert scale, instrument where lower scores represent better Communication and Teamwork, Interprofessional Learning, and Interprofessional Interactions. [14], and the Collaborative Behaviors Scale (CBS) [15].

### Program outputs

This single day event was conducted with nine faculty and 21 trainees. The nine faculty participants included 3 MPs, 2 ROs and 4 RTTs, and the 21 trainee participants included 6 RO residents (29%), 6 MP residents (29%), and 9 RTT students (43%), representing 5 clinical sites affiliated with the single academic institution. Six trainee participants were female (29%) and trainee participants were both junior (n = 5) radiation oncology residents postgraduate year (PGY) PGY1 (n = 1), RO residents PGY2 (n = 2), MP resident year 1 (n = 2) and senior, n = 16, RO residents PGY 3 (n = 3), MP year 2 (n = 4) and RTT year 3 (n = 9).

All participants completed three out of the five simulation scenarios as part of a team. All participants completed the pre and post event assessments (n = 42). All faculty completed post-case assessments (n = 9). All cases were completed within the allocated 105 minutes. Participants’ mean score was 9.1 (SD 1.2) when asked about their willingness to participate in future simulation events.

### Program outcomes

Prior to the event, 83% of RO residents and 83% of MP residents reported no or only occasional interaction with RTT trainees; 67% of RO and 100% of RTT trainees reported no or only occasional interaction with MP trainees and 56% of RTT trainees, and 33% of MP trainees reported no or only occasional interaction with RO trainees. The event increased participants’ exposure to other trainees; mean score ± SD was 8.5 ± 1.0 and increased their perception of the importance of interprofessional communication, with a mean score 9.1 ± 1.2. See Table 3 for the distribution of scores by trainee discipline.

**Table 3:** **Value of high-fidelity simulation to clinical education based on a 1 to 10 Likert Scale (1 = not valuable; 10 = extremely valuable)**

Aspect of education:	Radiation oncology	Medical physics	Radiation therapy
	Mean ± standard deviation	Mean ± standard deviation	Mean ± standard deviation
Clinical knowledge	8.7 ± 1.4	9.5 ± 0.8	8.6 ± 1.0
Clinical decision making	8.5 ± 1.0	9.2 ± 1.6	8.9 ± 1.3
Clinical skills	8.2 ± 1.3	9.7 ± 0.5	8.9 ± 0.9
Exposure to other trainees	7.8 ± 3.5	9.7 ± 0.8	8.1 ± 1.8
Interprofessional communication	8.7 ± 1.0	9.8 ± 0.4	9.0 ± 1.1

The mean score for self-reported improvements in clinical knowledge was 8.9 ± 1.1, for clinical decision making was 8.9 ± 1.3, and for clinical skills was 8.9 ± 1.1. The distribution of scores by discipline is in Table 3. Fifteen participants (71%) reported the complexity of the scenario was appropriate for their level of training and 6 (29%) participants reported the cases were too advanced. Three of 5 (60%) junior trainees reported the cases were too advanced and 3 of 16 (19%) senior trainees reported the cases were too advanced. No participants reported that the cases were too basic for this purpose.

All 16 (100%) participants who participated in the brachytherapy case reported that they had no exposure to a similar case scenario in their clinical training to date. Six of 16 (50%) participants in the electron case, 10 of the 11 (90%) participants in the pediatric case, 2 of 10 (20%) participants in the CBCT case and 4 of 10 (40%) participants in the Lung SBRT case reported no exposure to a similar case scenario in their clinical training to date. For each case, the mean (±SD) for how prepared they felt based on their current training was 5.4 ± 2.6 for brachytherapy, 7.2 ± 2.2 for electrons, 5.9 ± 1.7 for pediatric, 6.4 ± 1.8 for CBCT, and 6.3 ± 1.9 for Lung SBRT. Participants mean (±SD) for the perceived importance of exposure in training to each case was 9.3 ± 1.4 for brachytherapy, 9.5 ± 1.0 for electrons, 9.4 ± 1.1 for pediatric, 9.7 ± 0.7 for CBCT, and 9.0 ± 1.0 for Lung SBRT. Finally, the mean (±SD) for the perceived value of team interaction to patient care for each case was 8.5 ± 1.7 for brachytherapy, 9.2 ± 1.0 for electrons, 9.5 ± 0.7 for pediatric, 8.6 ± 1.6 for CBCT and 8.6 ± 1.5 for Lung SBRT.

With respect to interprofessional perceptions, participants averaged 83.5 and 85.2 respectively for pre- and post-event administrations of the RIPLS scale, and 60.6 and 55.7 for the UWEIQ scale. This reflected positive perceptions both pre- and post-event, and improvement in perceptions in both scales at the post-event administration. Group average scores for the CBS scale, which assessed collaborative behaviours in each case of the day, improved for all groups between the first and second case, decreasing slightly for most groups in the third case, presumably reflecting participant fatigue.

## Discussion

The practice and technology of radiation oncology have changed dramatically in the last two decades with, among other things, the incorporation of 3D planning, CBCT, integration of multiple imaging modalities including PET and stereotactic body radiotherapy, which typically involve more complicated processes and tasks [16]. The field of radiation oncology is expected to continue to evolve [17] and postgraduate training programs for RO, MP and RTT must adapt to keep pace with clinical practice [1]. In addition to evolving clinical content the practice of medicine is changing with greater emphasis on interprofessional teams [2, 18]. In CanMEDS 2015, the updated competency framework for physicians, the competencies of ‘effective teams’, ‘interprofessional heath care’ and patient quality and safety are explicitly defined as key concepts [18]. Also, the Institute of Medicine has emphasized the importance of effective teamwork in medical practice [19]. These updated training requirements echo the work by Frenk et al. [20] who articulate how health professional training program have failed to evolve and have created a gap between training and actual patient and population needs. Our study has shown that high-fidelity simulation is able to expose radiation oncology trainees to clinical situations to which they are not routinely exposed during clinical training and has demonstrated how important trainees feel exposure to such cases is to prepare them for clinical practice.

There are many publications addressing the impact of simulation on technical skill acquisition in surgical and anesthesiology training. While many report that simulation is feasible and some show improved skill in a randomized setting against other education methods, [21] the significant resource utilization associate with simulation requires careful consideration. Systematic reviews of simulation training have shown that simulation-based training can result in improved skills transfer to a clinical setting compared to those who do not receive simulation training [22]. However, simulation is a heterogeneous area and the intensity and duration of the simulation training as well as the validity of the assessment measures can impact the results of such studies. In radiation oncology, identification of the skills and clinical situations where high fidelity simulation may be beneficial is the initial step to further interrogation of the educational benefits. In our study, we tested the feasibility of 5 high acuity, low frequency scenarios where a traditional curriculum may not provide adequate exposure to complicated clinical processes. Our data suggest simulation is feasible and has high trainee satisfaction. The long-term educational impact requires further study.

Radiation medicine has unique interprofessional team interactions. In addition to improving skills acquisition, simulation offers an opportunity to teach and assess competencies such as teamwork, and interprofessional collaboration. However, there are challenges with interprofessional team training including addressing interprofessional content depth versus interprofessional learning objectives [2] and professional territoriality. Our study has demonstrated that radiation medicine trainees from RO, MP and RTT have limited clinical exposure to each other in traditional educational models. In this event all trainees identified team skill as essential for patient care. Hence, trainees must receive training in the manner in which they are expected to deliver clinical care [20] and this training will likely require educational methods such as simulation that adequately reflect the modern clinical context. Further work is needed to determine barriers to team training in radiation medicine.

The benefits of high-fidelity simulation training and team training in radiation medicine must be balanced with the costs of such events. Our data demonstrated significant expenditure of personnel resources, equipment, and consumables to conduct this simulation event. This was feasible in this context due to grant funding. Some costs associated with simulation training may be reduced with subsequent events, particularly those related to case development. Resource sharing among training programs may also reduce simulation event related costs. Since our data shows that the level of engagement decreased after the second scenario, shorter events could be envisioned. Further efforts in this area must balance the opportunity cost of a high-fidelity simulation event against other program activities. Careful case selection to ensure high educational impact and to address gaps in clinical training is essential. Caution must be exercised when using equipment in clinical use as there is potential for equipment damage, due to inexperience. Wherever possible, decommissioned equipment may be best to prevent any disruptions to clinical care if equipment is damaged. In addition, these cases represent complicated clinical scenarios and further work is needed to determine at which level of training the greatest educational impact would be provided for each professional group. Our data demonstrate that more junior trainees may require alternate case scenarios to more senior trainees.

## Conclusion

High-fidelity simulation is feasible in a radiation medicine context. However, such educational activities require significant resources, including personnel and equipment.
